# Time series analysis of temporal trends in the pertussis incidence in Mainland China from 2005 to 2016

**DOI:** 10.1038/srep32367

**Published:** 2016-08-31

**Authors:** Qianglin Zeng, Dandan Li, Gui Huang, Jin Xia, Xiaoming Wang, Yamei Zhang, Wanping Tang, Hui Zhou

**Affiliations:** 1 Department of Respiratory Medicine, Affiliated Hospital of Chengdu University, School of Clinical Medicine, Chengdu University, China; 2 Department of Laboratory Medicine, The Second Affiliated Hospital of Chongqing Medical University, China; 3 Central Laboratory, Affiliated Hospital of Chengdu University, School of Clinical Medicine, Chengdu University, China

## Abstract

Short-term forecast of pertussis incidence is helpful for advanced warning and planning resource needs for future epidemics. By utilizing the Auto-Regressive Integrated Moving Average (ARIMA) model and Exponential Smoothing (ETS) model as alterative models with R software, this paper analyzed data from Chinese Center for Disease Control and Prevention (China CDC) between January 2005 and June 2016. The ARIMA (0,1,0)(1,1,1)_12_ model (AICc = 1342.2 BIC = 1350.3) was selected as the best performing ARIMA model and the ETS (M,N,M) model (AICc = 1678.6, BIC = 1715.4) was selected as the best performing ETS model, and the ETS (M,N,M) model with the minimum RMSE was finally selected for in-sample-simulation and out-of-sample forecasting. Descriptive statistics showed that the reported number of pertussis cases by China CDC increased by 66.20% from 2005 (4058 cases) to 2015 (6744 cases). According to Hodrick-Prescott filter, there was an apparent cyclicity and seasonality in the pertussis reports. In out of sample forecasting, the model forecasted a relatively high incidence cases in 2016, which predicates an increasing risk of ongoing pertussis resurgence in the near future. In this regard, the ETS model would be a useful tool in simulating and forecasting the incidence of pertussis, and helping decision makers to take efficient decisions based on the advanced warning of disease incidence.

As a respiratory disease caused by Bordetella pertussis, pertussis (also known as whooping cough or 100-day cough) is one of the leading causes for serious illnesses in babies, children, teens, and adults. According to the 2015 revised *Chinese National Guidelines on Diagnosis and Management of Cough*, pertussis is considered as a common cause of prolonged cough illness in adolescents and adults while frequently being associated with other symptoms of whooping cough^1^. Although pertussis is recognized as one of the most common vaccine preventable diseases, yet it still causes nearly 300,000 deaths in children each year worldwide^2^. In United Kingdom, a high incidence rate of pertussis infection was reported in 37% of school-aged children between 2001 and 2006^2^. In United States, 48,277 cases of pertussis were reported in the most recent peak year of 2012 with many more cases still unreported^3^. In China, little is known about the occurrence of pertussis due to the scarcity of a large-scale investigation of the incidence of pertussis. A recent study from the capital of Shaanxi province which is located in the northwest of China, reported that only 5.31% of the confirmed pertussis cases were properly diagnosed, with a misdiagnosis rate as high as 94.69%^4^.

Accurate simulation and forecasting of the incidence of an infectious disease exert a significant impact on resource utilization and planning for future epidemics. However, epidemics of pertussis cannot be easily identified or managed, as some other respiratory pathogens often cause similar symptoms like pertussis. In addition, the change of disease incidence is influenced and constrained by changing trends, periodic changes, and random disturbances like other infectious diseases^5^. Therefore, it is necessary to identify some effective accurate disease forecasting models to predict disease incidence based on the historical data. Specially, time series analysis, which comprises methods for analysing time series data to extract meaningful statistics and other characteristics of the data, is naturally required in this field.

Time series analysis means to implement a model to predict future values based on the previously observed values. Time series analysis consists of several approaches like the Exponential Smoothing (ETS model), Auto-Regressive and Moving Average Model (ARMA model), Neural Network model and some other models are subject to intense methodological developments in recent years^6^,^7^,^8^,^9^. Some of these models, such as the ARIMA (or seasonal ARIMA) model and the ETS model taking both overall trends and seasonal changes into account were considered useful tools in modeling time series with trend, cyclicity and seasonality^10^,^11^. Accordingly, these models were appropriate for analyzing pertussis incidence in China. Inspired by the advantages of these models, these methods were considered to optimal for forecasting the pertussis incidence in mainland China.

## Materials and Methods

### Data resource

The data of pertussis was obtained from the Chinese Center for Disease Control and Prevention (China CDC) [http://www.chinacdc.cn/], and the Bulletin of the Ministry of Health (from China VIP database) [http://lib.cqvip.com/], and the data from January 2005 to June 2016 were assembled as monthly counts of the reported cases.

### Statistical Analysis

As the most well-known branch of decision supporting tools in clinical epidemiology, time series having being increasingly exploited in epidemiological research in recent years. Since ETS model and ARIMA model have become more popular in time series in recent years as mentioned above^9^,^12^, in this study, the two were implemented as the epidemiological analysis methods.

### ARIMA model

Originally proposed by Box and Jenkins in the early 1970s, ARIMA model has been viewed as one of the most effective models for describing and forecasting time series^13^,^14^. An ARIMA model contains three components: The “AR” stands for autoregressive, the “I” stands for Integration and the “MA” stands for moving average. Generally, some time series have some form of cyclicity or seasonality trends (i.e., yearly or monthly). To illustrate these periodically changed data, a seasonal ARIMA model is thus adopted for modeling such data sets. A seasonal ARIMA model would be designed as ARIMA (*p*,*d*,*q*)(*P*,*D*,*Q*)_s_, (*p* =  non-seasonal AR order, *d* = non-seasonal differencing, *q* = non-seasonal MA order, *P* = seasonal AR order, *D* = seasonal differencing, *Q* = seasonal MA order), and *s* = time span of repeating seasonal pattern. In R software, the seasonal ARIMA model automatically selected the parameters for the best performing model according to either the minimum of Akaike information criterion (AIC), the corrected Akaike information criterion (AICc) or the Bayesian information criterion (BIC)^15^,^16^. In the second step, the simulating and forecasting results are given by the chosen model. Finally, the Ljung-Box Q test was used to diagnose whether the residual error sequence was a white-noise sequence.

### ETS model

The ETS model considers the error, trend and seasonal components of a given time series and evaluates 30 possible alterative models prior to selecting the best performing model to simulate the data^17^. The major three parameters are the error, trend and seasonal components which can be additive (A), multiplicative (M) or none (N). The best performing model is chosen according to either the minimum of AIC, AICc or BIC. As an automatic forecasting model incorporating the foundations of exponential smoothing, the ETS technique provided the forecast package for the R software outlined by Hyndman^18^, the Ljung-Box Q test was also used to diagnose whether the residual error sequence was a white-noise sequence.

### The analytic procedure of pertussis incidence

In this paper, the analytic procedure of pertussis incidence in mainland China is divided into the following steps: The first step is the pre-processing step, to make the time series set stationary, and to choose the alternative models being considered, and to introduce the criteria that will be used to determine how well the alternative models performed. To remove short-term (monthly) fluctuations and determine the long term time-series over multiple years, the Hodrick-Prescott filter method is performed as the cyclical and seasonal decomposition method in this paper^19^,^20^,^21^. The ARCH-LM test is also provided to verify the existence of ARCH effects. The second step is the model processing step to perform in-sample simulating and forecasting, by running the “the “auto.arima()” code in R software, the best performing model ARIMA model with either the minimum of AIC, AICc, BIC is automatically selected, the Ljung-Box Q tests is performed to diagnose whether the residual error sequence the of the best performing models is white-noise sequence; For ETS modeling, the best performing ETS model is also automatically selected by following the principle of minimum of AIC, AICc or BIC. With the implementation of auto regressive conditional heteroscedasticity (ARCH) Lagrangian multiplier (LM) test, the structural break of the residuals is identified to determine the existence of volatility in the series. With testing goodness of in-sample simulating and forecasting, the optimal model with either the minimum of The Root Mean Square Error (RMSE), Mean Absolute Error (MAE), Mean Absolute Deviation (MAD) or Mean Absolute Percentage Error (MAPE) test is finally determined between the best performing ARIMA model and the best performing ETS model. The third step is to perform an out-of-sample forecasting with the optimal model.

The study was approved by the Affiliated Hospital of Chengdu University. As aggregated data with no personal information were involved. All statistical analysis is conducted through R software (version 3.2.3, The R Foundation for Statistical Computing, Vienna, Austria)^17^.

### Role of the funding and data sources

The opinions reported in this paper are those of the authors, which are independent from the funding sources, and no endorsement from China CDC or other official organizations is intended or should be inferred.

## Results

### General information

**Figure 1:**
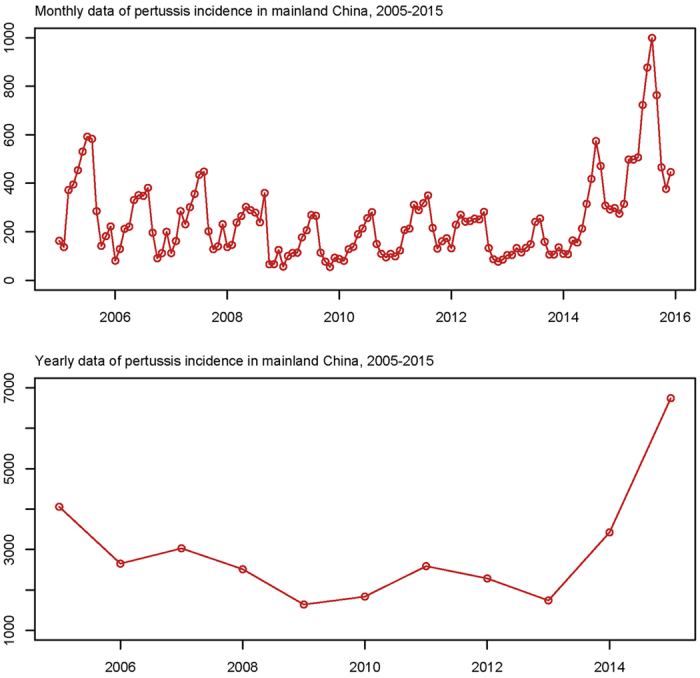
Yearly and monthly data of pertussis incidence numbers in mainland China from 2005 to 2015.

**Figure 2:**
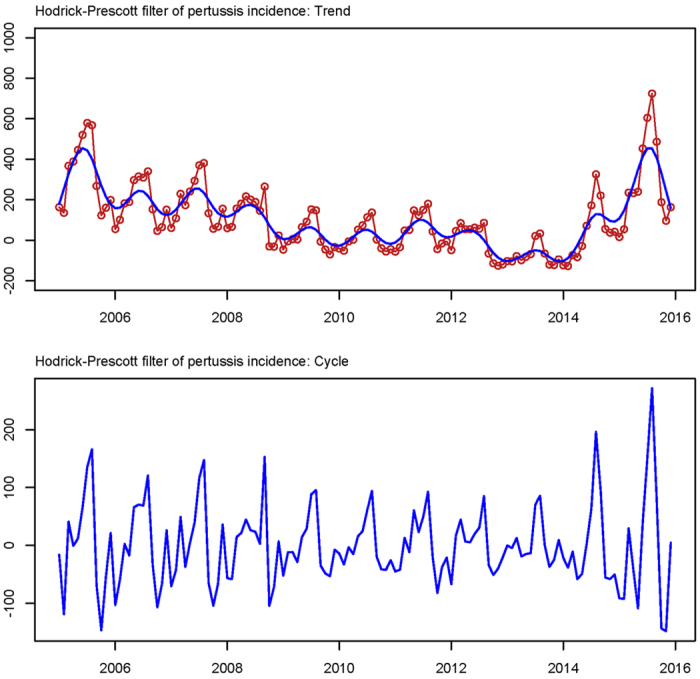
Results from decomposing the monthly pertussis incidence time series using the Hodrick-Prescott filter model with monthly smoothing parameter λ = 14,400.

According to the monthly reported incidence rates in China, no more than 10, 000 cases of pertussis are reported every year, which is much less than many other countries^22^, and the disease incidence is also much less than other infectious diseases like hand-foot-mouth disease in China^23^. In spite of the relative low incidence rates compared to other infectious disease, the incidence rates of pertussis had increased by 66.20%, from 4058 in 2005 to 6744 in 2015 in the last decade (Fig. 1). While using the Hodrick-Prescott filter method to remove short-term (monthly) fluctuations, a substantial rise was observed from 2013–2015, followed by a slight decrease from 2005–2013 (Fig. 2). In 2015, the reported number of cases reached 6744, with a 266.92% increase rate compared with the lowest number of cases reported in 2013 (1743 cases).

### In-sample simulating and forecasting

**Figure 3:**
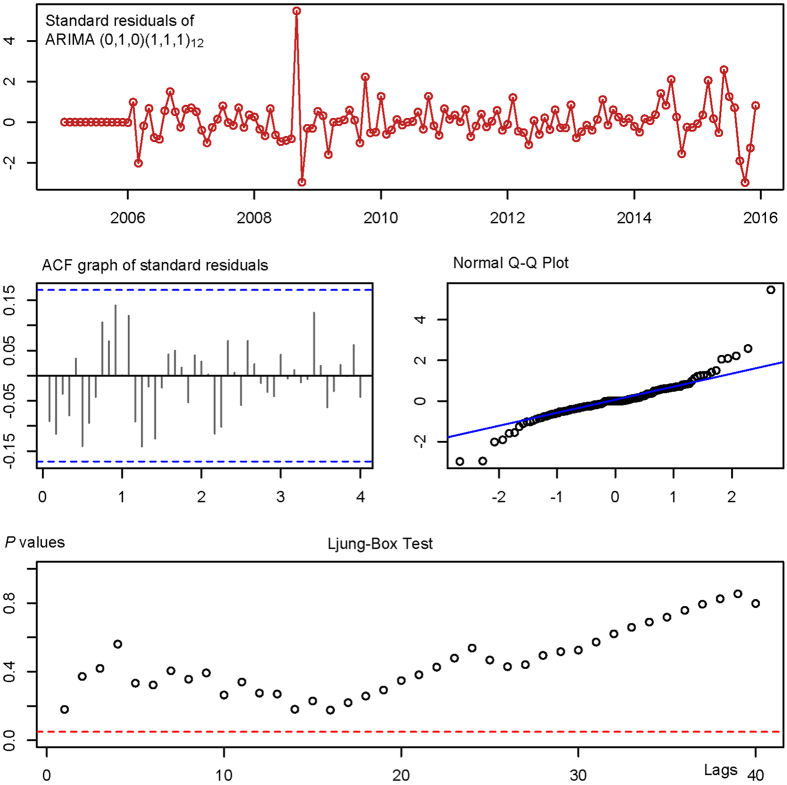
Goodness tests of in sample simulating and forecasting of ARIMA (0,1,0)(1,1,1)_[12]_ model.

**Figure 4:**
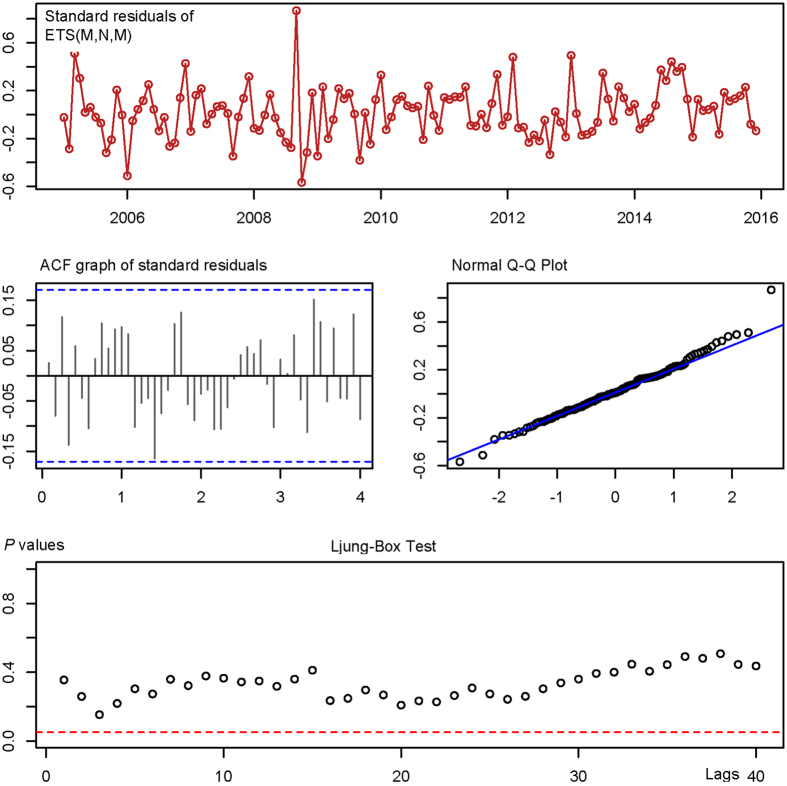
Goodness tests of in sample simulating and forecasting of ETS (M,N,M) model.

**Figure 5:**
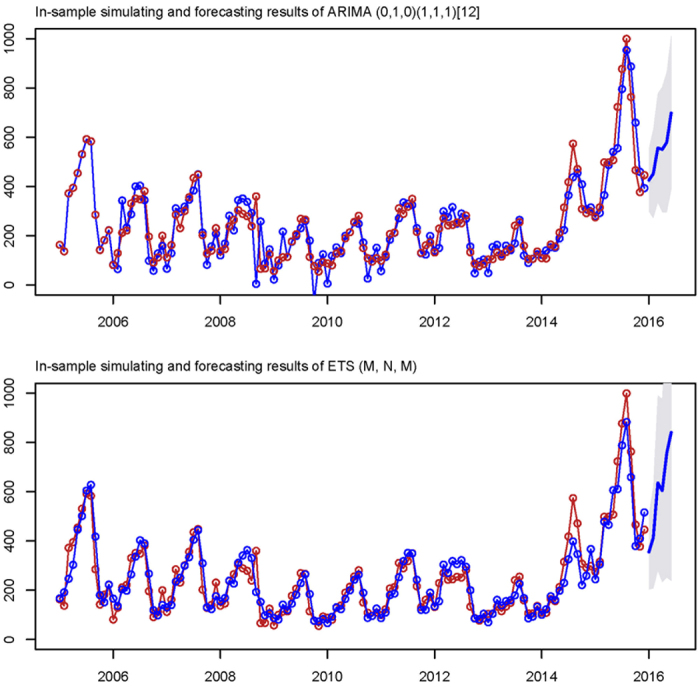
In-sample simulating and forecasting results of best performing models from each alterative model.

By running the auto.ARIMA code, the seasonal ARIMA (0,1,0)(1,1,1)_12_ model (AIC = 1341.99 AICc = 1342.2 BIC = 1350.3) was automatically selected as the best performing ARIMA model. According to its Ljung-Box Q test which was performed to assess the fitness of the ARIMA (0,1,0) × (1,1,1)_12_ model, the residual error sequence was closer to achieve white noise (*P*_Box-Ljung_>0.05 at 5, 10, 20, 30 and 40 lags, see Table 1 and Fig. 3); While running the ETS code, ETS (M,N,M) model (AIC = 1675.0, AICc = 1678.6, BIC = 1715.4) was automatically selected as the best performing ETS model, the Ljung-Box Q testing result of the ETS (M,N,M) model showed that the residual error sequence was closer to achieve white noise as well (*P*_Box-Ljung_>0.05 at 5, 10, 20, 30 and 40 lags, also see Table 1 and Fig. 4). By running the two best performing models, the in-sample-simulating and forecasting results were given as shown in Fig. 5. The ARCH-LM testing results in Table 2 showed the ARCH effect, which existed in the original series, was minimalized to a great extent in residuals of both the ARIMA (0,1,0) × (1,1,1)_12_ model and the and the ETS (M,N,M) model. In the step of goodness test of in-sample simulating and forecasting, under the principle of the minimum of RMSE, MAE, MAD or MAPE the ETS (M,N,M), the ETS (M,N,M) model (see Table 3), which was proved to provide more accurate forecasts, was finally chosen as the optimal model and was thus presented for out-of-sample forecasting.

### Out-of-sample forecasting

**Figure 6:**
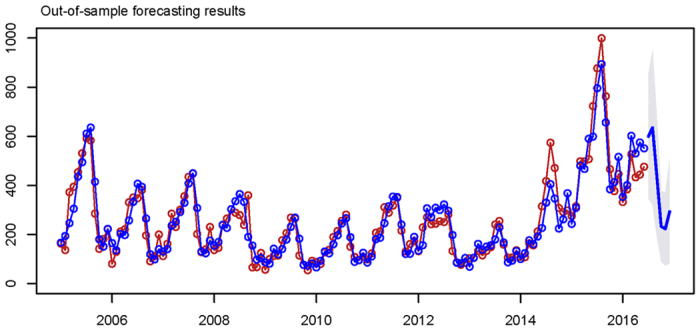
Out-of-sample forecasting results of pertussis incidence from July to December in 2016.

The forecasting results of pertussis incidence from July to December 2016 in mainland China were given by running the optimal ETS (M,N,M) model, Table 4 and Fig. 6 present the out-of-sample forecasting results from July to December 2016.

## Discussion

As a highly infectious respiratory illness, pertussis was a disease with high incidence rate before vaccination. However, with the development and widespread application of effective pertussis vaccines, dramatic changes happened in the epidemiology of pertussis globally^24^. In the United States, after the routine use of pertussis vaccine in 1943, the reported cases of pertussis dropped dramatically, and the number of cases was below 10, 000, which was the best in more than three decades (1965–2002). In China, pertussis immunization was introduced in the early 1960s, with three doses of whole-cell vaccine combined with diphtheria and tetanus toxoids (DTwP). Since 1982, a booster dose injected at 18–24 months has been added^4^, and the number of reported cases has dramatically decreased. However, in the recent resurgence of pertussis in many countries, a closer investigation on the aspects of pertussis causing its persistence should be prompted. Even with high vaccination rates, many countries have been reporting increasing pertussis cases in both the developed countries (the U.S, U.K, Netherlands, Portugal and Australia) and the developing countries (Cuba, Brazil, Mexico) in recent years^22^,^25^. Therefore, pertussis remains endemic worldwide which is still an important public health problem.

In this epidemiological study, the temporal trend of pertussis incidence in mainland China from 2005–2016 is analyzed according to the data reported by China CDC. Based on the descriptive statistics, it is observed that China has retained a lower pertussis reported cases from 2005 to 2013, which has been below 1 case per 100,000, lower than other countries^22^. In China, pertussis is always clinically diagnosed by physicians through laboratory methods such as culture and PCR, while serologic analysis is not commonly applied for diagnosis. Therefore, compared to other countries, its reported low incidence may be related to the adopted diagnostic criteria, suggesting substantial underreporting. Even though pertussis remains endemic to China, a sharp rise of reported numbers appeared in 2014 and 2015, which was a relatively high level. Whether an upward trend in incidence will be observed in 2016 is still unclear. Therefore, it is necessary to explore flexible and fractional methods for pertussis forecasting in a short term. In spite of time series models have been widely used in economics, environmental sciences and many other fields (eg. cerebrovascular diseases^13^, respiratory infections^26^,^27^, health care management, and so on^28^,^29^,^30^), know little about the flexibility of this model for time series analysis of the incidence of pertussis. Therefore, the requirement of this model is highlighted in this epidemiological study. Besides, an apparent cyclicity and seasonality was observed in the pertussis reporting. According to out-of-sample forecasting, the model forecasts a relatively high incidence cases in 2016, which predicates an increasing risk of ongoing pertussis resurgence in mainland China in the near future, indicating that pertussis never goes away completely^31^,^32^.

The seasonality of pertussis has been reported in some other countries as well^33^,^34^. Though some scholars have explored the mechanisms of pertussis incidence behind temporal information where the seasonal variations were captured with the autocorrelation analysis, the mechanism of pertussis activity in China remains unclear, which highlights a need to identify the factors for clarifying and explaining the cyclicity and seasonality^35^,^36^. In this study, the HP filter method was added to detect the cyclic and seasonal variation pertussis incidence, and a clear yearly cyclic pattern and seasonal pattern in the report of pertussis cases was found. During the study period, the peaks of seasonal periodicity occurred annually. For instance, the reported cases remained high in June until September. From January to February of the following year, the incidence was at a low level until the next reporting circle. The results of this study are consistent with previous studies, which showed an obvious cyclic and seasonal trend of the times series.

Globally, many countries are reporting the increase in pertussis cases, and the results of this study suggest an increasing risk of ongoing pertussis resurgence in mainland China in the near future, though the pertussis flare-up is unlikely to happen with the developed preventive systems for pertussis. Therefore, it is still important to continually remind that pertussis never goes away completely. In addition, effective efforts for controlling the potential pertussis resurgence should focus on professional recommendations, and appropriate public health education or instructions for people with high risk and enhancement of incident monitoring. From the methodological aspect, this study reveals that the selected ETS model is an assessable and flexible tool in forecasting the incidence of pertussis, and helping decision makers to provide advanced warning of future cases and further optimize distribution of resources based on the advanced warning of disease incidence.

## Additional Information

**How to cite this article**: Zeng, Q. *et al.* Time series analysis of temporal trends in the pertussis incidence in Mainland China from 2005 to 2016. *Sci. Rep.* **6**, 32367; doi: 10.1038/srep32367 (2016).

## Figures and Tables

**Table 1:** Ljung-Box Q tests of two best performing models.

ARIMA (0,1,0)(1,1,1)_12_			ETS(M,N,M)		
Lags	Chi-squared	*P* values	Lags	Chi-squared	*P* values
1	1.104	0.293	1	0.086	0.770
6	6.881	0.332	6	6.164	0.405
12	13.525	0.332	12	12.461	0.409
13	15.63	0.270	13	13.477	0.412
18	22.433	0.213	18	20.797	0.290
24	23.991	0.462	24	27.114	0.299
25	23.992	0.520	25	27.237	0.344
30	29.365	0.499	30	31.955	0.370

**Table 2:** Testing results of ARCH-effects about original series and best performing ARIMA and ETS models.

Original time series of pertussis incidence			Residual of the ARIMA (0,1,0) (1,1,1)_[12]_ model			Residual of the ETS(M,N,M) model		
Lags()	Chi-squared	*P* values	Lags()	Chi-squared	*P* values	Lags	Chi-squared	*P* values
1	92.467	<0.001	1	7.121	0.007	1	7.838	0.005
6	97.837	<0.001	6	7.939	0.243	6	10.225	0.116
12	98.818	<0.001	12	8.121	0.776	12	11.407	0.494
13	101.26	<0.001	13	8.744	0.792	13	13.281	0.426
18	98.714	<0.001	18	9.033	0.959	18	15.813	0.606
24	96.158	<0.001	24	9.173	0.997	24	17.548	0.824
25	95.253	<0.001	25	9.093	0.999	25	18.976	0.798
30	92.005	<0.001	30	9.966	0.999	30	18.957	0.941

**Table 3:** Parameter estimation for best performing ARIMA and ETS models for pertussis incidence.

Alterative models	ME	RMSE	*MAE*	*MPE*	*MAPE*	*MASE*
ARIMA (0,1,0)(1,1,1)_12_	2.900	61.689	39.151	−0.433	21.819	0.415
ETS(M,N,M) (Optimal model)	4.844	52.202	37.810	0.003	17.490	0.401

**Table 4:** Forecasting incidence cases of pertussis from July to December in 2016.

Time	Forecasts	95% CI
Jul-16	599	[345, 853]
Aug-16	635	[320, 950]
Sep-16	436	[192, 679]
Oct-16	232	[89, 376]
Nov-16	222	[73, 371]
Dec-16	293	[82, 504]
